# Fault-Tolerant Formation Tracking Control for Multi-Agent Systems with Bearing-Only Measurement

**DOI:** 10.3390/s26144500

**Published:** 2026-07-15

**Authors:** Yunjie Zhao, Jing Wang, Liya Dou, Meng Zhou, Shuang Ju

**Affiliations:** 1 School of Electrical and Control Engineering, North China University of Technology, Beijing 100144, China; 2 College of Information Science and Technology, Beijing University of Chemical Technology, Beijing 100029, China; 3 School of Electrical and Electronic Engineering, Shijiazhuang Tiedao University, Shijiazhuang 050043, China

**Keywords:** actuator fault, uncertainty, fault-tolerant control, formation realization, bearing measurement

## Abstract

This work investigates the formation control problem relying solely on bearing measurements for multi-agent systems in the two-dimensional plane. In order to achieve the desired geometric formation configuration between leaders and followers subject to actuator faults, this work first proposes a prescribed-time uncertainty observer that enables followers to estimate the uncertainties composed of unknown multiplicative and additive faults within the prescribed time. Then, relying on the uncertainty estimates and the maximum accelerations of the leaders, a bearing-only fault-tolerant formation control scheme is proposed for followers such that all agents asymptotically converge to the target formation. Agents are not required to exchange real-time dynamic information through communication. Furthermore, the stability of the closed-loop system is rigorously proved. Finally, simulation results verify the validity of the designed control schemes.

## 1. Introduction

Coordination control has captured extensive research focus in recent years. Through the collaboration of multiple agents, cooperative control enables the accomplishment of complex tasks and is widely used in areas including industry, transportation, military affairs and logistics [1,2]. Formation tracking control utilizing the leader–follower framework is a typical method for multi-agent cooperative control, which aims to make followers accurately track the leaders’ trajectories and maintain a preset geometric formation with them via the design of control strategies [3,4]. Formation control strategies can be classified into approaches relying on global position, relative position, distance, and bearing. The global positions of agents can be obtained through satellite positioning devices. The relative positions between agents can be determined using ultrasonic sensors and radio equipment [5]. Radar and visual sensors can measure the distance and bearing between agents, respectively [6].

Formation control relying on bearing measurements is a control strategy that only necessitates each agent to measure the relative angles of neighboring agents, without the requirement for relative position measurements, global position measurements, or distance measurements, thus offering the advantage of low economic cost [7,8]. With the leaders remaining stationary, control schemes based on angle measurements have been sequentially proposed for agents with different models, enabling all agents to achieve the preset formation geometry [9]. Considering the scenario where the constant speed magnitudes of the leaders are unavailable to the followers, algorithms that utilize angle measurements and rely on the leaders’ heading angles obtained through communication are proposed, enabling all agents to achieve the target formation [10,11,12]. To mitigate the unknown nonlinear disturbances acting on agents, robust formation control strategies relying on neighbor angular measurements and neighbor speed measurements are proposed, which enhances the stability of the system [13,14,15,16].

In practice, actuator faults including multiplicative faults and additive faults can make it difficult for the followers to achieve the preset formation geometry with the leaders. Therefore, the investigation of formation control problems with fault tolerance is imperative. To address the actuator fault problem of wheeled robots, adaptive algorithms are developed by utilizing the relative position and relative velocity measurements between robots, ensuring that all wheeled robots form a preset formation during movement [17]. In [18], an adaptive robust fault-tolerant algorithm is developed for actuator faults in spacecraft by utilizing the global position errors and attitude errors of the vehicles. For heterogeneous systems, algorithms with fault tolerance, which ensure that agents with diverse dynamic models can form and maintain the expected geometric formation structure, are presented in [19,20,21]. For actuator faults occurring in follower agents, a fault-tolerant containment formation control strategy relying on relative position error information is developed, which guarantees that all followers with actuator faults converge into the geometric containment region formed by the leaders [22]. Considering constant multiplicative faults and constant additive faults, fault observers have been proposed to effectively estimate the fault information of agents [23,24]. Through reinforcement learning, a control strategy designed for heterogeneous agents is proposed, enabling the followers with actuator faults of different models to effectively track a virtual leader [25]. Under the switching topology, fault-tolerant formation control methods relying on fuzzy logic have been proposed, which effectively compensates for actuator failures in agents and ensures the convergence and boundedness of system errors [26,27]. Taking multiplicative and additive faults as the uncertainties within agents, an observer utilizing the maximum uncertainty is developed for aerial vehicles [28]. For the fault problems of octocopters, an adaptive compensation control algorithm is introduced in [29].

However, the majority of fault-tolerant formation coordination methods for addressing actuator faults rely on the global or relative position errors of agents, with few studies reported on such control strategies based on bearing measurements. To achieve formation scaling of vehicles, an algorithm with fault tolerance is proposed, which only requires vehicles to measure the relative bearing and relative velocity of their neighbors [30]. When the leader is stationary, a bearing-only control scheme enables followers with actuator faults to achieve the expected geometric shape with the leaders within a predetermined time [31]. In scenarios where leaders move at variable speeds, a bearing-based fault-tolerant algorithm that depends on relative velocity measurements from neighboring agents addresses the issue of actuator faults in the agents [32].

In research on formation tracking control based on bearing measurements, existing works [10,11,12] require followers to acquire the variable heading angles of leaders through communication, whereas some studies [13,14,15,30,32] show that followers simultaneously measure the relative bearings and relative velocities of their neighboring agents. To address the source-seeking problem of quadrotors, a formation algorithm utilizing relative position and relative velocity between quadrotors and their neighbors is proposed, which enables multiple vehicles to move toward the source collectively [33]. However, harsh environments can cause agents to measure their neighbors’ velocities inaccurately, and electromagnetic interference can disrupt communication between agents. Based on the above discussion, this work investigates the fault-tolerant formation coordination issue for agents with actuator failures in the two-dimensional plane, in which agents only need to measure the bearings of their neighbors and do not require relative velocity measurements. Meanwhile, there is no necessity for agents to transmit dynamic information through communication. Given the unknown multiplicative faults and unknown additive faults of agents, a predefined-time uncertainty observer is developed to accurately assess the uncertainty composed of multiplicative and additive faults in the agents. Relying on the uncertainty estimates and the leaders’ maximum acceleration, a bearing-only fault-tolerant algorithm designed for followers is developed, enabling all followers with actuator faults to converge to the geometric shape of the formation as the leaders move at variable speeds. The formation control approach proposed in this work applies to unmanned ground vehicles and can tolerate actuator failures when these vehicles move in the two-dimensional plane. The contributions of this work are summarized as follows:

1. Compared with [23,24], which only accounts for constant multiplicative and additive faults in agents, this work considers time-varying multiplicative and additive faults.
2. Different from existing methods [17,18,19,20,21,22,23,24,25] requiring followers to measure global or relative positions, the proposed method only needs bearing measurement. Different from the fault-tolerant formation control methods [30,32] that require followers to measure both relative velocity and bearing of neighbors, the proposed method does not require relative velocity measurement for followers.
3. Compared with bearing-based formation control methods [10,11,12] that rely on communication, the proposed fault-tolerant formation scheme only depends on bearing measurement and requires no inter-agent communication.

The remaining part of this work is organized as follows: Section 2 introduces the notions and presents the problem statement of this work. A predefined-time uncertainty observer is developed in Section 3, and a bearing-only fault-tolerant algorithm is introduced in Section 4. Section 5 and Section 6 present the simulation results and conclusions of this work, respectively.

## 2. Preliminaries

### 2.1. Problem Formulation

Consider a set of agents, where $p_{i}\left(t\right)$ represents the position and $v_{i}\left(t\right)$ represents the velocity of agent *i*. A graph consisting of *n* agents and *m* edges is defined as G=(V,E), where V={1,2,⋯,n} denotes the set of nodes and E⊆V×V denotes the set of edges. In the graph *G*, the edge from agent *i* to agent *j* is defined as (i,j), which indicates that agent *i* can measure the relative angle of agent *j*. For the edge (i,j), agent *j* is defined as a neighbor of agent *i*, and the set of all neighbors of agent *i* is denoted as $N_{i}=\left\{j\in V:\left(i,j\right)\in E\right\}$. The graph *G* considered in this work is an undirected graph, which implies that (i,j)∈E⇔(j,i)∈E. The relative position vector $e_{ij}$ between agent *i* and agent *j* is defined as

(1) $$e_{ij}=p_{j}-p_{i}.$$

The relative bearing vector $g_{ij}$ between agents *i* and *j* is defined as

(2) $$g_{ij}=\frac{e_{ij}}{∥e_{ij}∥}.$$

**Figure 1:**
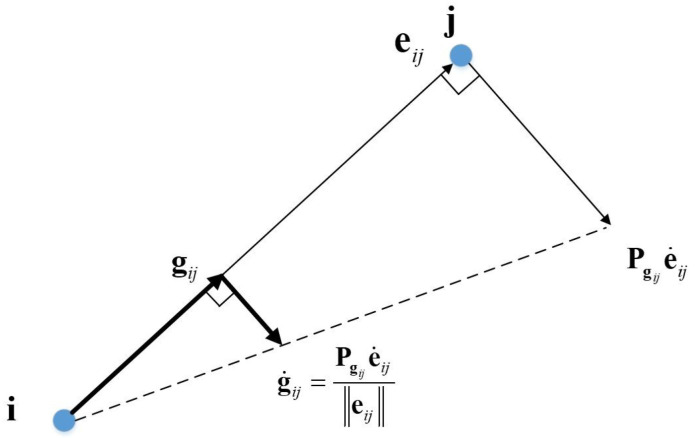
Geometric relationship between $g_{ij}$, $e_{ij}$ and ${\dot{g}}_{ij}$.

In a two-dimensional plane, $g_{ij}$ can also be described as $g_{ij}={\left[\cos\theta_{ij},\sin\theta_{ij}\right]}^{T}$, where $\theta_{ij}$ denotes the relative angle between agent *i* and agent *j*. The derivative of $g_{ij}$ is defined as

(3) $${\dot{g}}_{ij}=\frac{P_{g_{ij}}}{∥e_{ij}∥}{\dot{e}}_{ij},$$

where $P_{g_{ij}}=I_{2}-g_{ij}g_{ij}^{T}$ is a positive semi-definite matrix, and $I_{2}\in R^{2\times 2}$ denotes the identity matrix. Figure 1 illustrates the geometric relationship between $g_{ij}$, $e_{ij}$ and ${\dot{g}}_{ij}$ [9]. According to the geometric relationships in Figure 1, ${\dot{g}}_{ij}$ is orthogonal to both $g_{ij}$ and $e_{ij}$. Furthermore, we can derive that $g_{ij}^{T}{\dot{g}}_{ij}=0$ and $e_{ij}^{T}{\dot{g}}_{ij}=0$ [9].

Define the incidence matrix as $H\in R^{m\times n}$, where ${\left[H\right]}_{ki}=1$ indicates that the direction of the kth edge is towards agent *i*, and ${\left[H\right]}_{ki}=-1$ indicates that the direction of the kth edge is away from agent *i*. Let $e={\left[e_{1}^{T},e_{2}^{T},\cdots ,e_{m}^{T}\right]}^{T}$, $p={\left[p_{1}^{T},\cdots ,p_{n}^{T}\right]}^{T}$ and $\bar{H}=H⊗I_{2}$. We can obtain

(4) $$e=\bar{H}p.$$

Under the leader–follower framework, the *n* agents in graph *G* are divided into $n_{l}$ leaders and $n_{f}$ followers, where $n_{l}+n_{f}=n$. Agent $i,i\in \left\{1,2,\cdots ,n_{l}\right\}$ denotes a leader, and agent $i,i\in \left\{n_{l}+1,\cdots ,n_{l}+n_{f}\right\}$ denotes a follower. The bearing Laplacian matrix $B\in R^{2n\times 2n}$ is defined as [9]

(5) $${\left[B\right]}_{ij}=\left\{\begin{array}{c} 0_{2\times 2},i\neq j,\left(i,j\right)\notin E\\ -\left(I_{2}-g_{ij}^{*}{\left(g_{ij}^{*}\right)}^{T}\right),i\neq j,\left(i,j\right)\in E\\ \sum_{k\in N_{i}}\left(I_{2}-g_{ik}^{*}{\left(g_{ik}^{*}\right)}^{T}\right),i=j,i\in V \end{array}\right.$$

where ${\left[B\right]}_{ij}\in R^{2\times 2}$ represents the ij-th sub-block in matrix B. $I_{2}\in R^{2\times 2}$ denotes an identity matrix. $g_{ij}^{*}\in R^{2\times 1}$ represents the desired bearing vector between agent *i* and agent *j*. According to the division of agents into leaders and followers, the matrix B can be written as

(6) $$B=\left[\begin{array}{cc} B_{ll} & B_{lf}\\ B_{fl} & B_{ff} \end{array}\right],$$

where $B_{ll}$ denotes the interaction among leaders, and $B_{ff}$ represents the interaction among followers. Matrices $B_{lf}$ and $B_{fl}$ represent the interactions and bearing measurements between leaders and followers. The target formation utilizing angle measurements is defined as:

**Definition 1.**  A formation that satisfies the following conditions: (1) bearing: $g_{ij}=g_{ij}^{*},\forall \left(i,j\right)\in E$; (2) velocity: $v_{i}=v^{*},i\in \left\{n_{l}+1,\cdots ,n_{l}+n_{f}\right\}$ is defined as the target formation, where $v^{*}$ denotes the velocity of the leader.

**Figure 2:**
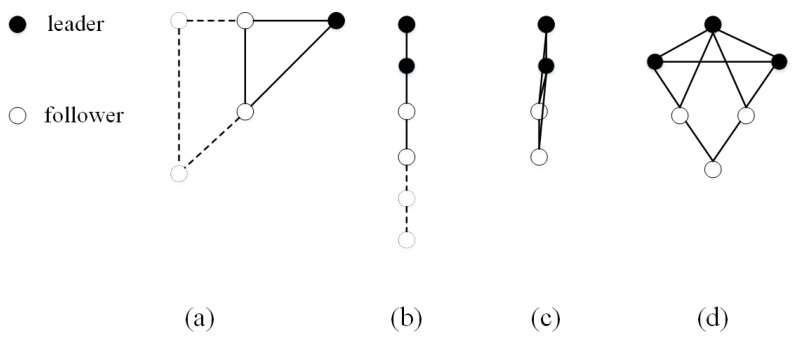
Unique and non-unique target formation using only bearing measurements. (**a**) Non-unique target formation; (**b**) non-unique target formation for collinear agents; (**c**) non-unique target formation induced by near-collinear arrangement of all agents, where visual sensors suffer occlusion and fail to acquire valid bearing measurements; (**d**) unique target formation.

It should be noted that when agents can only measure the relative angles of neighbors, the unique desired formation cannot be ensured by a single leader [9]. Additionally, the formation formed by all collinear agents is also not unique. Figure 2 illustrates unique and non-unique target formations based on bearing measurements. In Figure 2a, the followers’ desired positions cannot be uniquely determined due to a single leader, resulting in a non-unique target formation. There are two leaders in Figure 2b. Since all agents are collinear, the followers’ desired positions cannot be uniquely determined, which means the target formation is non-unique. Figure 2c illustrates the scenario where all agents are nearly collinear. Visual sensors are adopted to obtain inter-agent bearing information. When agents form near-collinear geometries, mutual occlusion occurs between each agent and its neighbors, which prevents valid bearing detection. Therefore, the target formation under near-collinear configurations is also non-unique in practice. There are three non-collinear leaders in Figure 2d, which avoids collinearity of all agents. The target formation based on bearing measurements is unique in this case. To guarantee that the desired target formation is unique, the following assumption is made.

**Assumption 1.** 
*There exist $n_{l}>2$ non-collinear leaders moving synchronously, and the matrix $B_{ff}$ is non-singular to ensure that the target formation is unique [9].*

**Remark 1.** 
*When the target formation is uniquely determined solely from angle measurements, the graph G is connected [11,12].*

**Remark 2.** 
Let $p_{l}={\left[p_{1}^{T},\cdots ,p_{n_{l}}^{T}\right]}^{T}$ denote the desired positions of the leaders. The desired positions of followers are denoted as $p_{f}^{*}$, where $p_{f}^{*}=-B_{ff}^{-1}B_{fl}p_{l}$. When matrix $B_{ff}$ is non-singular, it has an inverse matrix $B_{ff}^{-1}$, and the desired positions of followers can be uniquely determined.

**Remark 3.** 
*The collinear configuration in Figure 2b results in non-unique target formations. The near-collinear configuration in Figure 2c produces occlusions between agents, making it difficult to obtain bearing measurements. Multi-agent systems with collinear and near-collinear configurations are both unstable, and these two topologies should be avoided in practical implementations.*

### 2.2. Dynamic Description of Agents

The motion of leader $i,i\in \left\{1,2,\cdots ,n_{l}\right\}$ is given by

(7) $$\begin{array}{cc} \; & {\dot{p}}_{i}\left(t\right)=v_{i}\left(t\right)\\ \; & {\dot{v}}_{i}\left(t\right)=a_{i}\left(t\right),∥a_{i}\left(t\right)∥\leq a_{\max}, \end{array}$$

where $a_{i}\left(t\right)$ represents the acceleration of leader *i*, and $a_{\max}$ represents the leaders’ maximum acceleration. The motion of follower $i,i\in \left\{n_{l}+1,\cdots ,n_{l}+n_{f}\right\}$ with actuator faults is described as:

(8) $$\begin{array}{cc} \; & {\dot{p}}_{i}\left(t\right)=v_{i}\left(t\right)\\ \; & {\dot{v}}_{i}\left(t\right)=u_{i}^{F}\left(t\right)\\ \; & u_{i}^{F}\left(t\right)=\rho_{i}\left(t\right)u_{i}\left(t\right)+b_{i}\left(t\right), \end{array}$$

where $u_{i}^{F}\left(t\right)$ represents the control input of follower *i* under actuator fault conditions. $0<\rho_{i}\left(t\right)<1$ and $b_{i}\left(t\right)$ represent the unknown continuous multiplicative fault and the unknown continuous additive fault, respectively. $u_{i}\left(t\right)$ represents the faultless control input. In particular, $u_{i}^{F}\left(t\right)$ can be written as

(9) $$\begin{array}{cc} u_{i}^{F}\left(t\right) & =\rho_{i}\left(t\right)u_{i}\left(t\right)+b_{i}\left(t\right)\\ \; & =u_{i}\left(t\right)+\left(\rho_{i}-1\right)u_{i}\left(t\right)+b_{i}\left(t\right)\\ \; & =u_{i}\left(t\right)+D_{i}\left(t\right), \end{array}$$

where $D_{i}\left(t\right)=\left(\rho_{i}-1\right)u_{i}\left(t\right)+b_{i}\left(t\right)$ represents the uncertainty composed of multiplicative and additive faults of follower *i*. Define ${\hat{D}}_{i}\left(t\right)$ as the estimated uncertainty for follower *i*. The estimation error of uncertainty is defined as ${\tilde{D}}_{i}=D_{i}-{\hat{D}}_{i}$. To enable all followers with actuator faults to achieve the preset geometric shape of the formation alongside the leaders during movement, the problem statement of this work is given as follows:

**Problem 1.** 
*Under Assumption 1, design a prescribed-time uncertainty observer for each follower such that the estimated uncertainty ${\hat{D}}_{i}\left(t\right)$ approaches to the actual uncertainty $D_{i}\left(t\right)$ within a finite time.*

**Problem 2.** 
*Under Assumption 1, design a bearing-only fault-tolerant formation control scheme, enabling all followers with actuator faults and the leaders to achieve the expected geometric shape of the formation.*

**Assumption 2.** 
*For the uncertainty $D_{i}$ of follower i, there exists a positive constant $D_{i\max}$ such that $∥D_{i}∥\leq D_{i\max}$, where $D_{i\max}$ denotes the upper bound of the uncertainty for follower i [28,32].*

**Remark 4.** 
*The uncertainty $D_{i}$ consists of multiplicative faults and additive faults, which are constrained in practical engineering. Hence, Assumption 2 is reasonable [28,32].*

### 2.3. Nonsmooth Analysis

Consider the following differential equation:

(10) $$\dot{q}=g\left(q,t\right),$$

where *g* is a discontinuous function that is Lebesgue measurable. If the function *q* is absolutely continuous on $\left[t_{1},t_{2}\right]$ and satisfies the following differential inclusion:

(11) $$\dot{q}\in^{a.e}K\left[g\right]\left(q,t\right),$$

where $K\left[g\right]=\mathrm{co}\left\{\lim g\left(q_{i}\right)|q_{i}\to q\right\}$, the function *q* is called the Filippov solution of differential Equation (10) on the interval $\left[t_{1},t_{2}\right]$. Define a locally Lipschitz continuous function *V*, and the Clarke generalized gradient of the function *V* is defined as [34]:

(12) $$\partial V=co\left\{\lim\nabla V\left(q_{i}\right)|q_{i}\to q\right\}.$$

The set-valued Lie derivative of the function *V* relative to (10) is defined as [34]:

(13) $$\dot{\tilde{V}}≜\underset{\iota \in \partial V}{⋂}\iota^{T}K\left[g\right]\left(q,t\right).$$

According to the definitions of Filippov solutions and Clarke’s generalized gradient, all discontinuous points are removed and convex sets are defined; thus, there exists $\dot{V}\in^{a.e.}\dot{\tilde{V}}$ [34].

## 3. Prescribed-Time Uncertainty Observer

### 3.1. Prescribed-Time Control

To ensure that the uncertainty estimation errors of each follower converge within a predetermined time, a time-varying scaling function is defined as [7]

(14) $$\mu \left(t\right)=\left\{\begin{array}{c} \frac{T_{D}^{l}}{{\left(t_{0}+T_{D}-t\right)}^{l}},t\in \left[t_{0},t_{1}\right)\\ 1\;,t\in \left[t_{1},+\infty \right) \end{array}\right.$$

where $t_{0}$ and $t_{1}$ denote the starting time and the ending time, respectively, and $T_{D}=t_{1}-t_{0}$. l>0 is an adjustable parameter. The derivative of (14) is given by [7]

(15) $$\dot{\mu }\left(t\right)=\left\{\begin{array}{cc} \frac{l}{T_{D}}\mu^{1+\frac{1}{l}}, & t\in \left[t_{0},t_{1}\right)\\ 0, & t\in \left[t_{1},+\infty \right) \end{array}\right.$$

**Lemma 1.** 
*Consider the function $V:R\to R^{+}$. If there exist constants a>0 and b>0 such that $\dot{V}\leq -aV-b\frac{\dot{\mu }}{\mu }V$ in $t\in \left[t_{0},+\infty \right)$, then it follows that*

(16) $$V\left(t\right)\left\{\begin{array}{cc} \leq \mu^{-b}e^{-a\left(t-t_{0}\right)}V\left(t_{0}\right), & t\in \left[t_{0},t_{1}\right)\\ \equiv 0, & t\in \left[t_{1},+\infty \right) \end{array}\right.$$

*thus V is prescribed-time stable within time interval $T_{D}$ [7,8].*

### 3.2. Design of Prescribed-Time Uncertainty Observer

Define the velocity estimate of follower *i* as ${\hat{v}}_{i}$, and the velocity estimation error as $\delta_{i}=v_{i}-{\hat{v}}_{i}$. Define the estimate of $\delta_{i}$ as ${\hat{\delta }}_{i}$, and the estimation error of $\delta_{i}$ as ${\tilde{\delta }}_{i}=\delta_{i}-{\hat{\delta }}_{i}$. The prescribed-time uncertainty observer for follower $i,i\in \left\{n_{l}+1,n_{l}+2,\cdots ,n_{l}+n_{f}\right\}$ is constructed as follows

(17) $${\dot{\hat{v}}}_{i}=u_{i}+k_{1i}\delta_{i}+k_{2i}\int_{0}^{t}{\tilde{\delta }}_{i}\left(\tau \right)d\tau ,$$

(18) $${\hat{D}}_{i}=k_{1i}{\hat{\delta }}_{i}+{\dot{\delta }}_{i}+k_{2i}\int_{0}^{t}{\tilde{\delta }}_{i}\left(\tau \right)d\tau ,$$

(19) $${\dot{\hat{\delta }}}_{i}={\dot{\delta }}_{i}+\left(k_{3i}+k_{4i}\frac{\dot{\mu }}{\mu }\right){\tilde{\delta }}_{i}+k_{5i}\mathrm{sgn}\left({\tilde{\delta }}_{i}\right),$$

where $k_{1i}$, $k_{2i}$, $k_{3i}$, $k_{4i}$, and $k_{5i}$ are adjustable positive parameters.

**Theorem 1.** 
*Using the prescribed-time uncertainty observer (17)–(19), the estimation error of uncertainty ${\tilde{D}}_{i}$ for follower i can reach zero within the predetermined time $T_{D}$.*

**Proof.**  Construct the following Lyapunov function

(20) $$V_{i}=\frac{1}{2}{\tilde{\delta }}_{i}^{T}{\tilde{\delta }}_{i}.$$

The set-valued Lie derivative of Equation (20) is

(21) $${\dot{\tilde{V}}}_{i}={\tilde{\delta }}_{i}^{T}\left({\dot{\delta }}_{i}-{\dot{\hat{\delta }}}_{i}\right).$$

Substituting (19) into (21) yields

(22) $$\begin{array}{cc} {\dot{\tilde{V}}}_{i} & ={\tilde{\delta }}_{i}^{T}\left[{\dot{\delta }}_{i}-{\dot{\delta }}_{i}-\left(k_{3i}+k_{4i}\frac{\dot{\mu }}{\mu }\right){\tilde{\delta }}_{i}-k_{5i}K\left[\mathrm{sgn}\right]\left({\tilde{\delta }}_{i}\right)\right]\\ \; & ={\tilde{\delta }}_{i}^{T}\left[-\left(k_{3i}+k_{4i}\frac{\dot{\mu }}{\mu }\right){\tilde{\delta }}_{i}-k_{5i}K\left[\mathrm{sgn}\right]\left({\tilde{\delta }}_{i}\right)\right]\\ \; & =-\left(k_{3i}+k_{4i}\frac{\dot{\mu }}{\mu }\right){\tilde{\delta }}_{i}^{T}{\tilde{\delta }}_{i}-k_{5i}{\tilde{\delta }}_{i}^{T}K\left[\mathrm{sgn}\right]\left({\tilde{\delta }}_{i}\right). \end{array}$$

Since

(23) $$k_{5i}{\tilde{\delta }}_{i}^{T}K\left[\mathrm{sgn}\right]\left({\tilde{\delta }}_{i}\right)=k_{5i}{∥{\tilde{\delta }}_{i}∥}_{1},$$

We can obtain

(24) $${\dot{\tilde{V}}}_{i}=-\left(k_{3i}+k_{4i}\frac{\dot{\mu }}{\mu }\right){\tilde{\delta }}_{i}^{T}{\tilde{\delta }}_{i}-k_{5i}{∥{\tilde{\delta }}_{i}∥}_{1}.$$

Further, it can be deduced that

(25) $${\dot{\tilde{V}}}_{i}\leq -k_{3i}{\tilde{\delta }}_{i}^{T}{\tilde{\delta }}_{i}-k_{4i}\frac{\dot{\mu }}{\mu }{\tilde{\delta }}_{i}^{T}{\tilde{\delta }}_{i}.$$

Since ${\tilde{\delta }}_{i}^{T}{\tilde{\delta }}_{i}=2V_{i}$, (25) can be rewritten as

(26) $${\dot{\tilde{V}}}_{i}\leq -2k_{3i}V_{i}-2k_{4i}\frac{\dot{\mu }}{\mu }V_{i}.$$

According to Lemma 1 and inequality (26), when $t\geq T_{D}$, we have ${\tilde{\delta }}_{i}=0$. The uncertainty estimation error for follower *i* is

(27) $$\begin{array}{cc} {\tilde{D}}_{i} & =D_{i}-{\hat{D}}_{i}\\ \; & =D_{i}-k_{1i}{\hat{\delta }}_{i}-{\dot{\delta }}_{i}-k_{2i}\int_{0}^{t}{\tilde{\delta }}_{i}\left(\tau \right)d\tau \\ \; & =D_{i}-k_{1i}{\hat{\delta }}_{i}-\left({\dot{v}}_{i}-{\dot{\hat{v}}}_{i}\right)-k_{2i}\int_{0}^{t}{\tilde{\delta }}_{i}\left(\tau \right)d\tau . \end{array}$$

Since ${\dot{v}}_{i}=u_{i}+D_{i}$, substituting (17) into (27) yields

(28) $$\begin{array}{cc} {\tilde{D}}_{i} & =D_{i}-k_{1i}{\hat{\delta }}_{i}-\left[D_{i}-k_{1i}\delta_{i}-k_{2i}\int_{0}^{t}{\tilde{\delta }}_{i}\left(\tau \right)d\tau \right]-k_{2i}\int_{0}^{t}{\tilde{\delta }}_{i}\left(\tau \right)d\tau \\ \; & =k_{1i}\left(\delta_{i}-{\hat{\delta }}_{i}\right)\\ \; & =k_{1i}{\tilde{\delta }}_{i}. \end{array}$$

When $t\geq T_{D}$, the equation ${\tilde{\delta }}_{i}=0$ holds. Based on Equation (28), it can be further deduced that when $t\geq T_{D}$, ${\tilde{D}}_{i}=k_{1i}{\tilde{\delta }}_{i}=0$.

The proof is completed. □

## 4. Bearing-Only Fault-Tolerant Formation Control

Since followers (8) suffer from unknown continuous multiplicative and additive faults, traditional formation control methods cannot drive the bearing and velocity errors of the followers to converge. In Section 3, we propose an uncertainty estimation algorithm (17)–(19), which enables each follower to estimate the uncertainty composed of multiplicative and additive faults within a prescribed time. Furthermore, to ensure that the followers with actuator faults (8) and leaders (7) asymptotically converge to the target formation defined in Definition 1, it is necessary to design a fault-tolerant control scheme. Using the estimated uncertainty ${\hat{D}}_{i}$, the bearing-only fault-tolerant formation control law for follower $i,i\in \left\{n_{l}+1,n_{l}+2,\cdots ,n_{l}+n_{f}\right\}$ is given as follows

(29) $$\begin{array}{cc} u_{i}= & k_{a}\sum_{j\in N_{i}}{\dot{g}}_{ij}+k_{b}\sum_{j\in N_{i}}\left(g_{ij}-g_{ij}^{*}\right)\\ \; & +\sum_{j\in \{p,q,z\}}\left[\left(a_{\max}+∥{\hat{D}}_{i}∥+D_{i\max}\right)P_{g_{ij}}\eta_{i}^{T}\mathrm{sgn}\left(\eta_{i}{\dot{g}}_{ij}\right)\right]\\ \; & +k_{c}\sum_{j\in \{p,q,z\}}\left[P_{g_{ij}}\mathrm{sig}{\left({\dot{g}}_{ij}\right)}^{\alpha }\right]-{\hat{D}}_{i},\\ \eta_{i}= & {\left(P_{g_{ip}}+P_{g_{iq}}+P_{g_{iz}}\right)}^{-1}, \end{array}$$

where $k_{a}$, $k_{b}$, $k_{c}$ and α are adjustable positive gains. $g_{ij}$ and $g_{ij}^{*}$ denote the actual and desired bearing vectors between agent *i* and agent *j*, respectively. ${\dot{g}}_{ij}$ represents the derivative of the bearing vector, and $P_{g_{ij}}$ stands for the orthogonal projection matrix. $p,q,z\in \left\{1,2,\cdots ,n_{l}\right\}$, and *p*, *q*, *z* are non-collinear. $D_{i\max}$ denotes the upper bound of the uncertainty for follower *i*. Let $x\in R^{n\times 1}$, $x={\left[x_{1},x_{2},\cdots ,x_{n}\right]}^{T}$. sig(·) is defined as $\mathrm{sig}{\left(x\right)}^{\alpha }=\mathrm{sgn}\left(x\right){\left|x\right|}^{\alpha }$, where ${\left|x\right|}^{\alpha }={\left|x_{1}\right|}^{\alpha }+{\left|x_{2}\right|}^{\alpha }+\cdots +{\left|x_{n}\right|}^{\alpha }$. The design of the algorithm (29) solely relies on bearing measurements among agents, implying that each follower does not need to measure the relative velocity or the relative acceleration of neighboring agents. Meanwhile, none of the followers need to communicate with other agents to obtain their variable dynamic information. The design of the first, second and third terms on the right-hand side of control scheme (29) ensures that the bearing error of follower *i* converges to zero. The fourth term in control law (29) is utilized to adjust the convergence rate of bearing errors. The fifth term in (29) compensates for uncertainties of follower *i* caused by unknown multiplicative and additive faults. Since ${\dot{v}}_{i}=u_{i}+D_{i}$, one has

(30) $$\begin{array}{cc} {\dot{v}}_{i}= & k_{a}\sum_{j\in N_{i}}{\dot{g}}_{ij}+k_{b}\sum_{j\in N_{i}}\left(g_{ij}-g_{ij}^{*}\right)\\ \; & +\sum_{j\in \{p,q,z\}}\left[\left(a_{\max}+∥{\hat{D}}_{i}∥+D_{i\max}\right)P_{g_{ij}}\eta_{i}^{T}\mathrm{sgn}\left(\eta_{i}{\dot{g}}_{ij}\right)\right]\\ \; & +k_{c}\sum_{j\in \{p,q,z\}}\left[P_{g_{ij}}\mathrm{sig}{\left({\dot{g}}_{ij}\right)}^{\alpha }\right]+D_{i}-{\hat{D}}_{i}. \end{array}$$

Let $\gamma ={\left[v_{1}^{T},\cdots ,v_{n}^{T}\right]}^{T}$, $\gamma^{*}=1_{n}⊗v^{*}$, $g={\left[g_{1}^{T},\cdots ,g_{m}^{T}\right]}^{T}$, and $g^{*}={\left[{\left(g_{1}^{*}\right)}^{T},\cdots ,{\left(g_{m}^{*}\right)}^{T}\right]}^{T}$. Define the column vectors as $s_{i}=\sum_{j\in \{p,q,z\}}\left[\left(a_{\max}+∥{\hat{D}}_{i}∥+D_{i\max}\right)P_{g_{ij}}\eta_{i}^{T}\mathrm{sgn}\left(\eta_{i}{\dot{g}}_{ij}\right)\right]$ and $w_{i}=\sum_{j\in \{p,q,z\}}\left[P_{g_{ij}}\mathrm{sig}{\left({\dot{g}}_{ij}\right)}^{\alpha }\right]$. Define $S\in R^{2n\times 1}$, $W\in R^{2n\times 1}$, $Z\in R^{2n\times 1}$, and $Q\in R^{2n\times 1}$, where $S={\left[0,\cdots ,0,s_{n_{l}+1}^{T},\cdots ,s_{n_{l}+n_{f}}^{T}\right]}^{T}$, $W={\left[0,\cdots ,0,w_{n_{l}+1}^{T},\cdots ,w_{n_{l}+n_{f}}^{T}\right]}^{T}$, $Z={\left[0,\cdots ,0,{\left(D_{n_{l}+1}-{\hat{D}}_{n_{l}+1}\right)}^{T},\cdots ,{\left(D_{n_{l}+n_{f}}-{\hat{D}}_{n_{l}+n_{f}}\right)}^{T}\right]}^{T}$, and $Q={\left[{\dot{v}}_{1}^{T},\cdots ,{\dot{v}}_{n_{l}}^{T},0,\cdots ,0\right]}^{T}$. Let $v^{*}\in R^{2\times 1}$ represent the leader’s velocity. The dynamics of $n_{l}$ leaders and $n_{f}$ followers can be described in the following compact form

(31) $$\begin{array}{cc} \dot{\gamma }= & -k_{a}\left[\begin{array}{cc} 0_{2n_{l}\times 2n_{l}} & 0_{2n_{l}\times 2n_{f}}\\ 0_{2n_{f}\times 2n_{l}} & I_{2n_{f}\times 2n_{f}} \end{array}\right]{\bar{H}}^{T}\dot{g}-k_{b}\left[\begin{array}{cc} 0_{2n_{l}\times 2n_{l}} & 0_{2n_{l}\times 2n_{f}}\\ 0_{2n_{f}\times 2n_{l}} & I_{2n_{f}\times 2n_{f}} \end{array}\right]{\bar{H}}^{T}\left(g-g^{*}\right)\\ \; & +\;S+k_{c}W+Z+Q, \end{array}$$

where $I_{2n_{f}\times 2n_{f}}\in R^{2n_{f}\times 2n_{f}}$ is an identity matrix. The asymptotic stability of a system consisting of followers with faulty actuators and leaders moving with variable velocities can be stated by the subsequent theorem.

**Theorem 2.** 
*Using prescribed-time uncertainty observers (17)–(19) and a bearing-only fault-tolerant formation control law (29), all followers with actuator faults and leaders moving at variable velocities can approach the desired formation.*

**Proof.**  Construct the following Lyapunov function

(32) $$V=k_{b}e^{T}\left(g-g^{*}\right)+\frac{1}{2}{\left(\gamma -\gamma^{*}\right)}^{T}\left(\gamma -\gamma^{*}\right),$$

where $e^{T}\left(g-g^{*}\right)=\sum_{k=1}^{m}∥e_{k}∥\left(1-g_{k}^{T}g_{k}^{*}\right)\geq 0$. V=0 if and only if $g=g^{*}$ and $\gamma =\gamma^{*}$, which implies that all followers and all leaders have formed the target formation. The set-valued Lie derivative of (32) is

(33) $$\dot{\tilde{V}}=k_{b}e^{T}\dot{g}+k_{b}{\left(g-g^{*}\right)}^{T}\dot{e}+{\left(\gamma -\gamma^{*}\right)}^{T}\left(\dot{\gamma }-{\dot{\gamma }}^{*}\right).$$

Since $e^{T}\dot{g}=0$, $\dot{e}=\bar{H}\gamma$ and $\bar{H}\gamma^{*}=0$, it follows that

(34) $$\dot{\tilde{V}}=k_{b}{\left(g-g^{*}\right)}^{T}\bar{H}\left(\gamma -\gamma^{*}\right)+{\left(\gamma -\gamma^{*}\right)}^{T}\left(\dot{\gamma }-{\dot{\gamma }}^{*}\right).$$

Substituting (31) into the second term of (34), we have

(35) $$\begin{array}{cc} {\left(\gamma -\gamma^{*}\right)}^{T}\left(\dot{\gamma }-{\dot{\gamma }}^{*}\right)= & -\;k_{a}{\left(\gamma -\gamma^{*}\right)}^{T}\left[\begin{array}{cc} 0_{2n_{l}\times 2n_{l}} & 0_{2n_{l}\times 2n_{f}}\\ 0_{2n_{f}\times 2n_{l}} & I_{2n_{f}\times 2n_{f}} \end{array}\right]{\bar{H}}^{T}\dot{g}\\ \; & -\;k_{b}{\left(\gamma -\gamma^{*}\right)}^{T}\left[\begin{array}{cc} 0_{2n_{l}\times 2n_{l}} & 0_{2n_{l}\times 2n_{f}}\\ 0_{2n_{f}\times 2n_{l}} & I_{2n_{f}\times 2n_{f}} \end{array}\right]{\bar{H}}^{T}\left(g-g^{*}\right)\\ \; & +\;{\left(\gamma -\gamma^{*}\right)}^{T}S+k_{c}{\left(\gamma -\gamma^{*}\right)}^{T}W+{\left(\gamma -\gamma^{*}\right)}^{T}Z\\ \; & +\;{\left(\gamma -\gamma^{*}\right)}^{T}Q-{\left(\gamma -\gamma^{*}\right)}^{T}{\dot{\gamma }}^{*}. \end{array}$$

Since

(36) $${\left(\gamma -\gamma^{*}\right)}^{T}\left[\begin{array}{cc} 0_{2n_{l}\times 2n_{l}} & 0_{2n_{l}\times 2n_{f}}\\ 0_{2n_{f}\times 2n_{l}} & I_{2n_{f}\times 2n_{f}} \end{array}\right]={\left(\gamma -\gamma^{*}\right)}^{T},$$

it can be obtained that

(37) $$\begin{array}{cc} {\left(\gamma -\gamma^{*}\right)}^{T}\left(\dot{\gamma }-{\dot{\gamma }}^{*}\right) & =-k_{a}{\left(\gamma -\gamma^{*}\right)}^{T}{\bar{H}}^{T}\dot{g}-k_{b}{\left(\gamma -\gamma^{*}\right)}^{T}{\bar{H}}^{T}\left(g-g^{*}\right)\\ \; & +\;{\left(\gamma -\gamma^{*}\right)}^{T}S+k_{c}{\left(\gamma -\gamma^{*}\right)}^{T}W+{\left(\gamma -\gamma^{*}\right)}^{T}Z\\ \; & +\;{\left(\gamma -\gamma^{*}\right)}^{T}Q-{\left(\gamma -\gamma^{*}\right)}^{T}{\dot{\gamma }}^{*}. \end{array}$$

Given that ${\left(\gamma -\gamma^{*}\right)}^{T}Q=0$, one has

(38) $$\begin{array}{cc} {\left(\gamma -\gamma^{*}\right)}^{T}\left(\dot{\gamma }-{\dot{\gamma }}^{*}\right) & =-k_{a}{\left(\gamma -\gamma^{*}\right)}^{T}{\bar{H}}^{T}\dot{g}-k_{b}{\left(\gamma -\gamma^{*}\right)}^{T}{\bar{H}}^{T}\left(g-g^{*}\right)\\ \; & +\;{\left(\gamma -\gamma^{*}\right)}^{T}S+k_{c}{\left(\gamma -\gamma^{*}\right)}^{T}W+{\left(\gamma -\gamma^{*}\right)}^{T}Z\\ \; & -\;{\left(\gamma -\gamma^{*}\right)}^{T}{\dot{\gamma }}^{*}. \end{array}$$

The third term of Equation (38) can be written as

(39) $$\begin{array}{cc} \; & {\left(\gamma -\gamma^{*}\right)}^{T}S\\ \; & =\sum_{i=n_{l}+1}^{n_{l}+n_{f}}\left\{\left(a_{\max\;}+∥{\hat{D}}_{i}∥+D_{i\max\;}\right){\left(v_{i}-v^{*}\right)}^{T}\sum_{j\in \{p,q,z\}}\left[P_{g_{ij}}\eta_{i}^{T}K\left[\mathrm{sgn}\right]\left(\eta_{i}{\dot{g}}_{ij}\right)\right]\right\}\\ \; & =\sum_{i=n_{l}+1}^{n_{l}+n_{f}}\left\{\begin{array}{c} \left(a_{\max\;}+∥{\hat{D}}_{i}∥+D_{i\max\;}\right)\\ {\left(v_{i}-v^{*}\right)}^{T}\sum_{j\in \{p,q,z\}}\left[P_{g_{ij}}\eta_{i}^{T}K\left[\mathrm{sgn}\right]\left(\eta_{i}\frac{P_{g_{ij}}}{∥e_{ij}∥}\left(v^{*}-v_{i}\right)\right)\right] \end{array}\right\}\\ \; & =-\sum_{i=n_{l}+1}^{n_{i}+n_{f}}\left\{\begin{array}{c} \left(a_{\max\;}+∥{\hat{D}}_{i}∥+D_{i\max\;}\right)\\ \sum_{j\in \{p,q,z\}}\left[{\left(v_{i}-v^{*}\right)}^{T}P_{g_{ij}}^{T}\eta_{i}^{T}K\left[\mathrm{sgn}\right]\left(\eta_{i}P_{g_{ij}}\left(v_{i}-v^{*}\right)\right)\right] \end{array}\right\}, \end{array}$$

where $P_{g_{ij}}=P_{g_{ij}}^{T}$. Given that

(40) $${\left(v_{i}-v^{*}\right)}^{T}P_{g_{ij}}^{T}\eta_{i}^{T}K\left[\mathrm{sgn}\right]\left(\eta_{i}P_{g_{ij}}\left(v_{i}-v^{*}\right)\right)={∥\eta_{i}P_{g_{ij}}\left(v_{i}-v^{*}\right)∥}_{1},$$

Equation (39) can be written as

(41) $${\left(\gamma -\gamma^{*}\right)}^{T}S=-\sum_{i=n_{l}+1}^{n_{l}+n_{f}}\left\{\left(a_{\max\;}+∥{\hat{D}}_{i}∥+D_{i\max}\right)\sum_{j\in \{p,q,z\}}{∥\eta_{i}P_{g_{ij}}\left(v_{i}-v^{*}\right)∥}_{1}\right\}.$$

For follower *i*, $P_{g_{ij}}$ is

(42) $$\begin{array}{cc} P_{g_{ij}} & =I_{2}-g_{ij}g_{ij}^{T}\\ \; & =\left[\begin{array}{cc} 1-\cos^{2}\theta_{ij} & -\sin\theta_{ij}\cos\theta_{ij}\\ -\sin\theta_{ij}\cos\theta_{ij} & 1-\sin^{2}\theta_{ij} \end{array}\right], \end{array}$$

and the determinant of $P_{g_{ip}}+P_{g_{iq}}+P_{g_{iz}}$ is

(43) $$\begin{array}{cc} \; & \det\left(\sum_{j\in \{p,q,z\}}P_{g_{ij}}\right)\\ \; & ={\left(\cos\theta_{ip}\sin\theta_{iq}-\cos\theta_{iq}\sin\theta_{ip}\right)}^{2}+{\left(\cos\theta_{ip}\sin\theta_{iz}-\cos\theta_{iz}\sin\theta_{ip}\right)}^{2}\\ \; & +{\left(\cos\theta_{iq}\sin\theta_{iz}-\cos\theta_{iz}\sin\theta_{iq}\right)}^{2}. \end{array}$$

Since *p*, *q*, *z* are non-collinear, ${\left(\cos\theta_{ip}\sin\theta_{iq}-\cos\theta_{iq}\sin\theta_{ip}\right)}^{2}$, ${\left(\cos\theta_{ip}\sin\theta_{iz}-\cos\theta_{iz}\sin\theta_{ip}\right)}^{2}$, and ${\left(\cos\theta_{iq}\sin\theta_{iz}-\cos\theta_{iz}\sin\theta_{iq}\right)}^{2}$ cannot all be zero simultaneously, $\det\left(\sum_{j\in \{p,q,z\}}P_{g_{ij}}\right)\neq 0$. Furthermore, it can be deduced that the matrix $P_{g_{ip}}+P_{g_{iq}}+P_{g_{iz}}$ has an inverse matrix $\eta_{i}$. Since

(44) $$\sum_{j\in \{p,q,z\}}{∥\eta_{i}P_{g_{ij}}\left(v_{i}-v^{*}\right)∥}_{1}\geq {∥\sum_{j\in \{p,q,z\}}\left[\eta_{i}P_{g_{ij}}\left(v_{i}-v^{*}\right)\right]∥}_{1},$$

(45) $$\begin{array}{cc} \sum_{j\in \{p,q,z\}}\left[\eta_{i}P_{g_{ij}}\left(v_{i}-v^{*}\right)\right] & ={\left(P_{g_{ip}}+P_{g_{iq}}+P_{g_{iz}}\right)}^{-1}\left(P_{g_{ip}}+P_{g_{iq}}+P_{g_{iz}}\right)\left(v_{i}-v^{*}\right)\\ \; & =v_{i}-v^{*}, \end{array}$$

it can be deduced that

(46) $$\sum_{j\in \{p,q,z\}}{∥\eta_{i}P_{g_{ij}}\left(v_{i}-v^{*}\right)∥}_{1}\geq {∥v_{i}-v^{*}∥}_{1},$$

(47) $$-\sum_{j\in \{p,q,z\}}{∥\eta_{i}P_{g_{ij}}\left(v_{i}-v^{*}\right)∥}_{1}\leq -{∥v_{i}-v^{*}∥}_{1},$$

(48) $$\begin{array}{cc} \; & -\sum_{i=n_{l}+1}^{n_{l}+n_{f}}\left\{\left(a_{\max\;}+∥{\hat{D}}_{i}∥+D_{i\max\;}\right)\sum_{j\in \{p,q,z\}}{∥\eta_{i}P_{g_{ij}}\left(v_{i}-v^{*}\right)∥}_{1}\right\}\\ \; & \leq -\sum_{i=n_{l}+1}^{n_{l}+n_{f}}\left\{\left(a_{\max\;}+∥{\hat{D}}_{i}∥+D_{i\max\;}\right){∥v_{i}-v^{*}∥}_{1}\right\}, \end{array}$$

(49) $${\left(\gamma -\gamma^{*}\right)}^{T}S\leq -\sum_{i=n_{l}+1}^{n_{l}+n_{f}}\left\{\left(a_{\max\;}+∥{\hat{D}}_{i}∥+D_{i\max\;}\right){∥v_{i}-v^{*}∥}_{1}\right\}.$$

The fourth term of Equation (38) can be rewritten as

(50) $$\begin{array}{cc} \; & \;k_{c}{\left(\gamma -\gamma^{*}\right)}^{T}W\\ \; & =k_{c}\sum_{i=n_{l}+1}^{n_{l}+n_{f}}\left\{{\left(v_{i}-v^{*}\right)}^{T}\sum_{j\in \{p,q,z\}}\left[P_{g_{ij}}\mathrm{sig}{\left({\dot{g}}_{ij}\right)}^{\alpha }\right]\right\}\\ \; & =k_{c}\sum_{i=n_{l}+1}^{n_{l}+n_{f}}\left\{{\left(v_{i}-v^{*}\right)}^{T}\sum_{j\in \{p,q,z\}}\left[P_{g_{ij}}K\left[\mathrm{sgn}\right]\left({\dot{g}}_{ij}\right){\left|{\dot{g}}_{ij}\right|}^{\alpha }\right]\right\}\\ \; & =k_{c}\sum_{i=n_{l}+1}^{n_{l}+n_{f}}\left\{{\left(v_{i}-v^{*}\right)}^{T}\sum_{j\in \{p,q,z\}}\left[P_{g_{ij}}K\left[\mathrm{sgn}\right]\left(\frac{P_{g_{ij}}}{∥e_{ij}∥}\left(v^{*}-v_{i}\right)\right){\left|{\dot{g}}_{ij}\right|}^{\alpha }\right]\right\}\\ \; & =-k_{c}\sum_{i=n_{l}+1}^{n_{l}+n_{f}}\left\{\sum_{j\in \{p,q,z\}}\left[{\left(v_{i}-v^{*}\right)}^{T}P_{g_{ij}}^{T}K\left[\mathrm{sgn}\right]\left(P_{g_{ij}}\left(v_{i}-v^{*}\right)\right){\left|{\dot{g}}_{ij}\right|}^{\alpha }\right]\right\}. \end{array}$$

where $P_{g_{ij}}=P_{g_{ij}}^{T}$. As

(51) $${\left(v_{i}-v^{*}\right)}^{T}P_{g_{ij}}^{T}K\left[\mathrm{sgn}\right]\left(P_{g_{ij}}\left(v_{i}-v^{*}\right)\right)={∥P_{g_{ij}}\left(v_{i}-v^{*}\right)∥}_{1},$$

we can obtain

(52) $$k_{c}{\left(\gamma -\gamma^{*}\right)}^{T}W=-k_{c}\sum_{i=n_{l}+1}^{n_{l}+n_{f}}\left\{\sum_{j\in \{p,q,z\}}\left({∥P_{g_{ij}}\left(v_{i}-v^{*}\right)∥}_{1}{\left|{\dot{g}}_{ij}\right|}^{\alpha }\right)\right\}.$$

The fifth term of Equation (38) can be rewritten as:

(53) $$\begin{array}{cc} {\left(\gamma -\gamma^{*}\right)}^{T}Z & =\sum_{i=n_{l}+1}^{n_{l}+n_{f}}\left[{\left(v_{i}-v^{*}\right)}^{T}\left(D_{i}-{\hat{D}}_{i}\right)\right]\\ \; & \leq \sum_{i=n_{l}+1}^{n_{l}+n_{f}}\left(\left(∥{\hat{D}}_{i}∥+D_{i\max}\right){∥v_{i}-v^{*}∥}_{1}\right). \end{array}$$

The sixth term of Equation (38) can be rewritten as:

(54) $$\begin{array}{cc} -{\left(\gamma -\gamma^{*}\right)}^{T}{\dot{\gamma }}^{*} & =-\sum_{i=n_{l}+1}^{n_{l}+n_{f}}\left[{\left(v_{i}-v^{*}\right)}^{T}{\dot{v}}^{*}\right]\\ \; & \leq \sum_{i=n_{l}+1}^{n_{l}+n_{f}}\left(a_{\max}{∥v_{i}-v^{*}∥}_{1}\right). \end{array}$$

Substituting (49), (52)–(54) into (38) yields

(55) $$\begin{array}{cc} {\left(\gamma -\gamma^{*}\right)}^{T}\left(\dot{\gamma }-{\dot{\gamma }}^{*}\right)\leq & -k_{a}{\left(\gamma -\gamma^{*}\right)}^{T}{\bar{H}}^{T}\dot{g}-k_{b}{\left(\gamma -\gamma^{*}\right)}^{T}{\bar{H}}^{T}\left(g-g^{*}\right)\\ \; & -\;k_{c}\sum_{i=n_{l}+1}^{n_{l}+n_{f}}\left\{\sum_{j\in \{p,q,z\}}\left({∥P_{g_{ij}}\left(v_{i}-v^{*}\right)∥}_{1}{\left|{\dot{g}}_{ij}\right|}^{\alpha }\right)\right\}\\ \; & -\;\sum_{i=n_{l}+1}^{n_{l}+n_{f}}\left(\left(a_{\max\;}+∥{\hat{D}}_{i}∥+D_{i\max\;}\right){∥v_{i}-v^{*}∥}_{1}\right)\\ \; & +\;\sum_{i=n_{l}+1}^{n_{l}+n_{f}}\left(\left(a_{\max\;}+∥{\hat{D}}_{i}∥+D_{i\max\;}\right){∥v_{i}-v^{*}∥}_{1}\right). \end{array}$$

Furthermore, we can obtain

(56) $$\begin{array}{cc} {\left(\gamma -\gamma^{*}\right)}^{T}\left(\dot{\gamma }-{\dot{\gamma }}^{*}\right) & \leq -k_{a}{\left(\gamma -\gamma^{*}\right)}^{T}{\bar{H}}^{T}\dot{g}-k_{b}{\left(\gamma -\gamma^{*}\right)}^{T}{\bar{H}}^{T}\left(g-g^{*}\right)\\ \; & -\;k_{c}\sum_{i=n_{l}+1}^{n_{l}+n_{f}}\left\{\sum_{j\in \{p,q,z\}}\left({∥P_{g_{ij}}\left(v_{i}-v^{*}\right)∥}_{1}{\left|{\dot{g}}_{ij}\right|}^{\alpha }\right)\right\}. \end{array}$$

Substituting (56) into (34) yields

(57) $$\begin{array}{cc} \dot{\tilde{V}} & \leq -k_{a}{\left(\gamma -\gamma^{*}\right)}^{T}{\bar{H}}^{T}\dot{g}-k_{c}\sum_{i=n_{l}+1}^{n_{l}+n_{f}}\left\{\sum_{j\in \{p,q,z\}}\left({∥P_{g_{ij}}\left(v_{i}-v^{*}\right)∥}_{1}{\left|{\dot{g}}_{ij}\right|}^{\alpha }\right)\right\}\\ \; & \leq 0, \end{array}$$

where $-k_{a}{\left(\gamma -\gamma^{*}\right)}^{T}{\bar{H}}^{T}\dot{g}=-k_{a}{\dot{e}}^{T}\dot{g}=-k_{a}\sum_{k=1}^{m}{\dot{e}}_{k}^{T}{\dot{g}}_{k}=-k_{a}\sum_{k=1}^{m}{\dot{e}}_{k}^{T}\frac{P_{gk}}{∥e_{k}∥}{\dot{e}}_{k}\leq 0$. Substituting (38) into (34) yields

(58) $$\begin{array}{cc} \dot{\tilde{V}}= & -k_{a}{\left(\gamma -\gamma^{*}\right)}^{T}{\bar{H}}^{T}\dot{g}+{\left(\gamma -\gamma^{*}\right)}^{T}S+k_{c}{\left(\gamma -\gamma^{*}\right)}^{T}W\\ \; & +\;{\left(\gamma -\gamma^{*}\right)}^{T}Z-{\left(\gamma -\gamma^{*}\right)}^{T}{\dot{\gamma }}^{*}. \end{array}$$

Equation (38) can be rewritten as

(59) $$\begin{array}{cc} {\left(\gamma -\gamma^{*}\right)}^{T}\left(\dot{\gamma }-{\dot{\gamma }}^{*}\right) & =-k_{a}{\left(\gamma -\gamma^{*}\right)}^{T}{\bar{H}}^{T}\dot{g}+k_{b}\sum_{i=1}^{n_{l}+n_{f}}\left[v_{i}^{T}\sum_{j\in N_{i}}\left(g_{ij}-g_{ij}^{*}\right)\right]\\ \; & +\;{\left(\gamma -\gamma^{*}\right)}^{T}S+k_{c}{\left(\gamma -\gamma^{*}\right)}^{T}W+{\left(\gamma -\gamma^{*}\right)}^{T}Z-{\left(\gamma -\gamma^{*}\right)}^{T}{\dot{\gamma }}^{*}. \end{array}$$

According to lasalle’s invariance principle, the system errors converge to a set when $\dot{\tilde{V}}=0$. From Equation (58), it can be deduced that $\dot{\tilde{V}}=0\Rightarrow \gamma =\gamma^{*}$. According to Equation (59), when $\gamma =\gamma^{*}$, we can derive that $g_{ij}=g_{ij}^{*},\forall \left(i,j\right)\in E$. At this time, the lyapunov function (32) satisfies V=0. All followers and leaders have formed the desired geometric formation.

The proof is completed. □

**Remark 5.** 
*Considering the asymptotic stability of the closed-loop system [35], the proof of Theorem 2 indicates that both the bearing errors and velocity errors of the multi-agent system converge asymptotically to zero, and all agents can asymptotically reach the target formation.*

**Remark 6.** 
*According to (57) and (58), the parameters $k_{a}$ and $k_{c}$ affect the convergence rate of the Lyapunov function (32). As the parameters $k_{a}$ and $k_{c}$ increase, the value of $|\dot{V}|$ rises, and the convergence rate of the Lyapunov function (32) becomes faster.*

**Remark 7.** 
The right-hand side of the closed-loop dynamic Equation (31) satisfies the conditions of lebesgue measurability and local essential boundedness. According to the classical existence theorem for Filippov solutions [36], there necessarily exists an absolutely continuous solution (γ(t),g(t)) defined on the interval $\left[0,T^{*}\right)$, where $T^{*}\in \left(0,+\infty \right]$. The derivations of Equations (32)–(57) hold at any point where the solution exists, which yields $\dot{\tilde{V}}\left(t\right)\leq 0,\forall t\in \left[0,T^{*}\right)$. Furthermore, we can conclude that V(t)≤V(0) holds for all $t\in \left[0,T^{*}\right)$. In (32), $e^{T}\left(g-g^{*}\right)$ stands for the bearing errors of all agents, and ${\left(\gamma -\gamma^{*}\right)}^{T}\left(\gamma -\gamma^{*}\right)$ stands for the velocity errors of all agents. Given V(t)≤V(0), it can be concluded that the bearing g(t) and velocity γ(t) of all agents are bounded over the interval $\left[0,T^{*}\right)$. Based on the Filippov solution extension theorem [37] and the boundedness of g(t) and γ(t), it can be deduced that the solution to the closed-loop system exists globally on [0,+∞).

To further address the collision avoidance problem in bearing-based formation control schemes, we present collision avoidance conditions among agents. Let V(0)=α and $\beta =\frac{2∥\bar{H}∥}{\lambda_{\min\;}\left(B_{\mathrm{ff}}\right)}$. Define the desired minimum distance between any two agents as $\min_{i,j\in V}∥p_{i}^{*}-p_{j}^{*}∥$. Let $0<d<\min_{i,j\in V}∥p_{i}^{*}-p_{j}^{*}∥$. Collision avoidance among agents can be described by the following corollary.

**Corollary 1.** 
Under Assumption 1, if the positive constants α, β and d satisfy $\frac{\alpha \beta +\sqrt{\alpha^{2}\beta^{2}+4\alpha \beta ∥{\tilde{p}}^{*}∥}}{2}\leq \frac{1}{\sqrt{n}}\left(\min_{i,j\in V}∥p_{i}^{*}-p_{j}^{*}∥-d\right)$, then $∥p_{i}\left(t\right)-p_{j}\left(t\right)∥\geq d$ for all i,j∈V and all t≥0.

**Proof.**  Define the desired position of each agent as $p_{i}^{*}\left(t\right)$, and the geometric centroid of the target formation as ${\bar{p}}^{*}\left(t\right)=\left[\sum_{i=1}^{n}p_{i}^{*}\left(t\right)\right]/n$. Let $p^{*}\left(t\right)={\left[{\left(p_{1}^{*}\left(t\right)\right)}^{T},\cdots ,{\left(p_{n}^{*}\left(t\right)\right)}^{T}\right]}^{T}$, ${\tilde{p}}^{*}\left(t\right)=p^{*}\left(t\right)-1_{n}⊗{\bar{p}}^{*}\left(t\right)$ and $p\left(t\right)={\left[{\left(p_{1}\left(t\right)\right)}^{T},{\left(p_{2}\left(t\right)\right)}^{T},\cdots ,{\left(p_{n}\left(t\right)\right)}^{T}\right]}^{T}$. The bearing laplacian matrix can be written in the following form

(60) $$B={\bar{H}}^{T}D\left(P_{g_{k}^{*}}\right)\bar{H},$$

where $D\left(P_{g_{k}^{*}}\right)=\mathrm{blk}\mathrm{diag}\left(P_{g_{1}^{*}},\cdots ,P_{g_{m}^{*}}\right)$. Hence, we have

(61) $$\begin{array}{cc} p^{T}Bp & =p^{T}{\bar{H}}^{T}D\left(P_{g_{k}^{*}}\right)\bar{H}p\\ \; & =e^{T}D\left(P_{g_{k}^{*}}\right)e\\ \; & =\sum_{k=1}^{m}{∥e_{k}∥}^{2}\left[1-{\left(g_{k}^{T}g_{k}^{*}\right)}^{2}\right]\\ \; & =\sum_{k=1}^{m}{∥e_{k}∥}^{2}\left(1-g_{k}^{T}g_{k}^{*}\right)\left(1+g_{k}^{T}g_{k}^{*}\right)\\ \; & \leq 2\max_{k}∥e_{k}∥\sum_{k=1}^{m}∥e_{k}∥\left(1-g_{k}^{T}g_{k}^{*}\right)\\ \; & =2\max_{k}∥e_{k}∥e^{T}\left(g-g^{*}\right). \end{array}$$

Furthermore, we can derive that

(62) $$\frac{p^{T}Bp}{2\max∥e_{k}∥}\leq e^{T}\left(g-g^{*}\right).$$

Define $\delta_{p}\left(t\right)=p\left(t\right)-p^{*}\left(t\right)$, we can derive $\delta_{p}\left(t\right)={\left[0^{T},\delta_{pf}^{T}\left(t\right)\right]}^{T}$, $\delta_{p}^{T}B\delta_{p}=\delta_{pf}^{T}B_{ff}\delta_{pf}$, and ${\mathrm{Bp}}^{*}=0$, where $\delta_{pf}={\left[\delta_{p_{n_{l}+1}}^{T},\cdots ,\delta_{p_{n_{l}+n_{f}}}^{T}\right]}^{T}$. This implies

(63) $$\begin{array}{cc} \max_{k}∥e\left(k\right)∥ & \leq \;∥e∥\;=\;∥\bar{H}p∥\;=\;∥\bar{H}\left(p-p^{*}+p^{*}\right)∥\\ \; & \leq \;∥\bar{H}\delta_{p}∥+∥\bar{H}p^{*}∥\\ \; & =\;∥\bar{H}\delta_{p}∥+∥\bar{H}p^{*}-\bar{H}\left(1_{n}⊗{\bar{p}}^{*}\right)∥\\ \; & =\;∥\bar{H}\delta_{p}∥+∥\bar{H}{\tilde{p}}^{*}∥\\ \; & \leq \;∥\bar{H}∥\left(∥\delta_{p}∥+∥{\tilde{p}}^{*}∥\right), \end{array}$$

(64) $$p^{T}\mathrm{Bp}=\delta_{p}^{T}B\delta_{p}\geq \lambda_{\min}\left(B_{ff}\right){∥\delta_{pf}∥}^{2}=\lambda_{\min}\left(B_{ff}\right){∥\delta_{p}∥}^{2}.$$

Let V(0)=α, Since the set-valued Lie derivative of equation $\dot{\tilde{V}}\leq 0$, we can deduce that V≤α. Inequality (62) can be rewritten as

(65) $$\frac{\lambda_{\min}\left(B_{ff}\right){∥\delta_{p}∥}^{2}}{2∥\bar{H}∥\left(∥\delta_{p}∥+∥{\tilde{p}}^{*}∥\right)}\leq e^{T}\left(g-g^{*}\right)\leq \alpha .$$

Let $\beta =\frac{2∥\bar{H}∥}{\lambda_{\min}\left(B_{ff}\right)}$, Inequality (65) can be rewritten as

(66) $$\frac{1}{\beta }\frac{{∥\delta_{p}∥}^{2}}{\left(∥\delta_{p}∥+∥{\tilde{p}}^{*}∥\right)}\leq \alpha .$$

Therefore, we can obtain that $∥\delta_{p}∥\in \left[0,\frac{\alpha \beta +\sqrt{\alpha^{2}\beta^{2}+4\alpha \beta ∥{\tilde{p}}^{*}∥}}{2}\right]$. Since $p_{i}\left(t\right)-p_{j}\left(t\right)=\left[p_{i}\left(t\right)-p_{i}^{*}\left(t\right)\right]-\left[p_{j}\left(t\right)-p_{j}^{*}\left(t\right)\right]+\left[p_{i}^{*}\left(t\right)-p_{j}^{*}\left(t\right)\right]$, the distance between agents *i* and *j* can be described as

(67) $$\begin{array}{cc} ∥p_{i}\left(t\right)-p_{j}\left(t\right)∥ & \geq ∥p_{i}^{*}\left(t\right)-p_{j}^{*}\left(t\right)∥-∥p_{i}\left(t\right)-p_{i}^{*}\left(t\right)∥-∥p_{j}\left(t\right)-p_{j}^{*}\left(t\right)∥\\ \; & \geq ∥p_{i}^{*}\left(t\right)-p_{j}^{*}\left(t\right)∥-\sum_{k=1}^{n}∥p_{k}\left(t\right)-p_{k}^{*}\left(t\right)∥\\ \; & \geq ∥p_{i}^{*}\left(t\right)-p_{j}^{*}\left(t\right)∥-\sqrt{n}∥p\left(t\right)-p^{*}\left(t\right)∥\\ \; & =∥p_{i}^{*}\left(t\right)-p_{j}^{*}\left(t\right)∥-\sqrt{n}∥\delta_{p}\left(t\right)∥. \end{array}$$

When $\frac{\alpha \beta +\sqrt{\alpha^{2}\beta^{2}+4\alpha \beta ∥{\tilde{p}}^{*}∥}}{2}\leq \frac{1}{\sqrt{n}}\left(\min_{i,j\in V}∥p_{i}^{*}-p_{j}^{*}∥-d\right)$, we can deduce that $∥\delta_{p}∥\in \left[0,\frac{1}{\sqrt{n}}\left(\min_{i,j\in V}∥p_{i}^{*}-p_{j}^{*}∥-d\right)\right]$. Furthermore, we have

(68) $$\begin{array}{cc} ∥p_{i}\left(t\right)-p_{j}\left(t\right)∥ & =∥p_{i}^{*}\left(t\right)-p_{j}^{*}\left(t\right)∥-\sqrt{n}∥\delta_{p}\left(t\right)∥\\ \; & \geq ∥p_{i}^{*}\left(t\right)-p_{j}^{*}\left(t\right)∥-\min_{i,j\in V}∥p_{i}^{*}-p_{j}^{*}∥+d\\ \; & >0. \end{array}$$

Inequality (68) implies that no collisions occur among all agents during the process of converging to the target formation.

The proof is completed. □

## 5. Numerical Simulations

**Figure 3:**
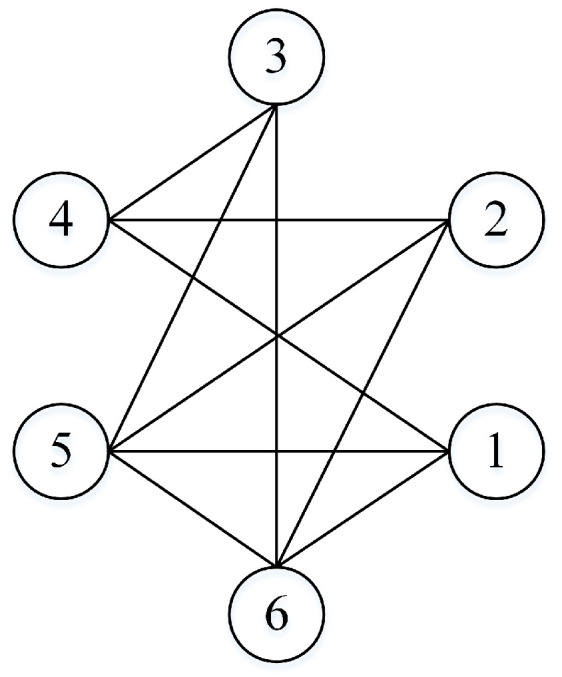
Topological structure.

Consider the graph *G* consisting of six second-order agents. Agent i,i∈{1,2,3} denotes the leader, and agent i,i∈{4,5,6} denotes the follower with an actuator fault. The desired angles between the agents are set to $\theta_{41}^{*}=-\pi /6$, $\theta_{42}^{*}=0$, $\theta_{43}^{*}=\pi /6$, $\theta_{51}^{*}=0$, $\theta_{52}^{*}=\pi /6$, $\theta_{53}^{*}=\pi /3$, $\theta_{56}^{*}=-\pi /6$, $\theta_{61}^{*}=\pi /6$, $\theta_{62}^{*}=\pi /3$, $\theta_{63}^{*}=\pi /2$, $\theta_{65}^{*}=5\pi /6$. Figure 3 illustrates the topological structure among the agents.

### 5.1. Numerical Simulations for the Bearing-Only Fault-Tolerant Formation Control

The speed of the leader is set to $v^{*}\left(t\right)=c^{*}\left(t\right){\left[\cos\theta^{*}\left(t\right),\sin\theta^{*}\left(t\right)\right]}^{T}$, where $c^{*}\left(t\right)=1+0.9\cos\left(0.25t\right)$ and $\theta^{*}\left(t\right)=0.6+0.8\cos\left(0.25t\right)$. The maximum acceleration of the leaders is set to $a_{\max}=2\;m/s^{2}$. The multiplicative faults of the followers are set to $\rho_{4}\left(t\right)=0.7+0.3\sin\left(0.6t\right)\cos\left(0.6t\right)$, $\rho_{5}\left(t\right)=0.5+0.4\cos^{2}\left(0.6t\right)$, $\rho_{6}\left(t\right)=0.8+0.5\sin\left(0.5t\right)\cos\left(0.5t\right)$. The additive faults of the followers are set to $b_{4}\left(t\right)={\left[b_{4x}\left(t\right),b_{4y}\left(t\right)\right]}^{T}$, $b_{5}\left(t\right)={\left[b_{5x}\left(t\right),b_{5y}\left(t\right)\right]}^{T}$, $b_{6}\left(t\right)={\left[b_{6x}\left(t\right),b_{6y}\left(t\right)\right]}^{T}$, where

$$b_{4x}\left(t\right)=\left\{\begin{array}{c} 0.7\sin(0.4t),0\leq t\leq 40\\ 0.5202t-21.0075,40\leq t\leq 50\\ -0.3t+20,50\leq t\leq 60\\ 0.4t-22,60\leq t\leq 70\\ -0.4t+34,70\leq t\leq 80 \end{array};\right.$$

$$b_{4y}\left(t\right)=\left\{\begin{array}{c} 0.8\cos(0.6t),0\leq t\leq 40\\ 0.2661t-10.3035,40\leq t\leq 50\\ -0.8t+43,50\leq t\leq 60\\ 0.9t-59,60\leq t\leq 70\\ -0.9t+67,70\leq t\leq 80 \end{array};\right.$$

$$b_{5x}\left(t\right)=1+1.2\sin\left(0.3t\right);$$

$$b_{5y}\left(t\right)=1+1.4\cos\left(0.5t\right);$$

$$b_{6x}\left(t\right)=-3+1.7\sin\left(0.5t\right);$$

$$b_{6y}\left(t\right)=-3+1.3\cos\left(0.6t\right).$$

The upper bounds of uncertainties for the followers are set as $D_{4\max}=10$, $D_{5\max}=10$, $D_{6\max}=10$. The parameters of the time-varying scaling function are set as l=1, $T_{D}=10$ s. The parameters of the uncertainty observer for follower 4 are set as $k_{14}=5$, $k_{24}=3.2$, $k_{34}=5$, $k_{44}=1$, and $k_{54}=0.5$. The parameters of the uncertainty observer for follower 5 are selected as $k_{15}=6$, $k_{25}=3$, $k_{35}=4$, $k_{45}=2$, and $k_{55}=0.8$. The parameters of the uncertainty observer for follower 6 are set as $k_{16}=7$, $k_{26}=4$, $k_{36}=6$, $k_{46}=3$ and $k_{56}=2$. The parameters of the bearing-only fault-tolerant formation control law (29) are set as $k_{a}=1.6$, $k_{b}=40$, $k_{c}=2$, α=1. The initial velocities of the followers are set to $v_{4}\left(0\right)={\left[v_{4x}\left(0\right),v_{4y}\left(0\right)\right]}^{T}$, $v_{5}\left(0\right)={\left[v_{5x}\left(0\right),v_{5y}\left(0\right)\right]}^{T}$ and $v_{6}\left(0\right)={\left[v_{6x}\left(0\right),v_{6y}\left(0\right)\right]}^{T}$, where $v_{4x}\left(0\right)$,$v_{4y}\left(0\right)$,$v_{5x}\left(0\right)$,$v_{5y}\left(0\right)$,$v_{6x}\left(0\right)$,$v_{6y}\left(0\right)$ are random numbers in the interval [−2,2].

**Figure 4:**
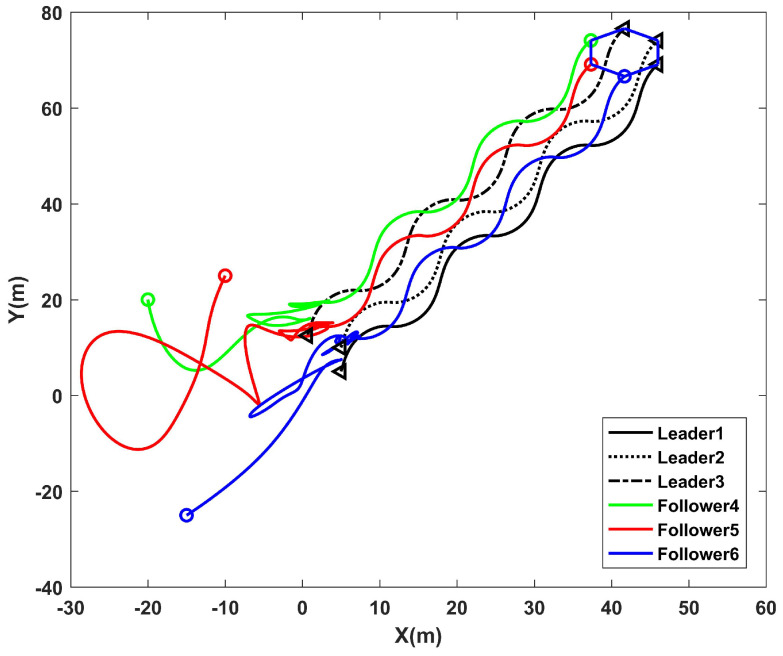
Trajectories of all leaders and all followers.

**Figure 5:**
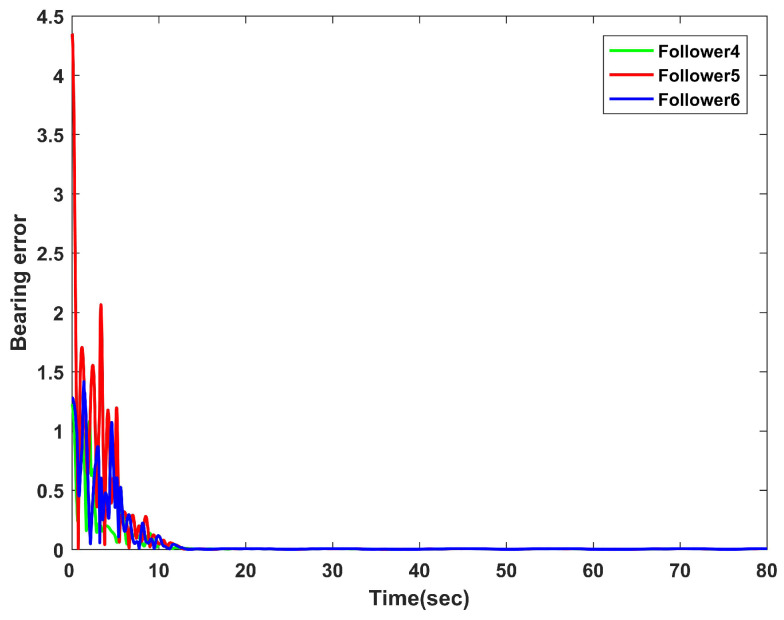
Follower agents’ bearing error.

**Figure 6:**
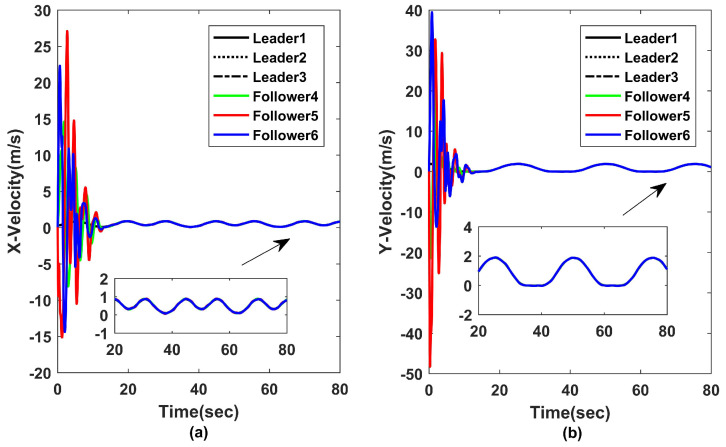
Velocities of all leaders and all followers: (**a**) x-axis velocities; (**b**) y-axis velocities.

**Figure 7:**
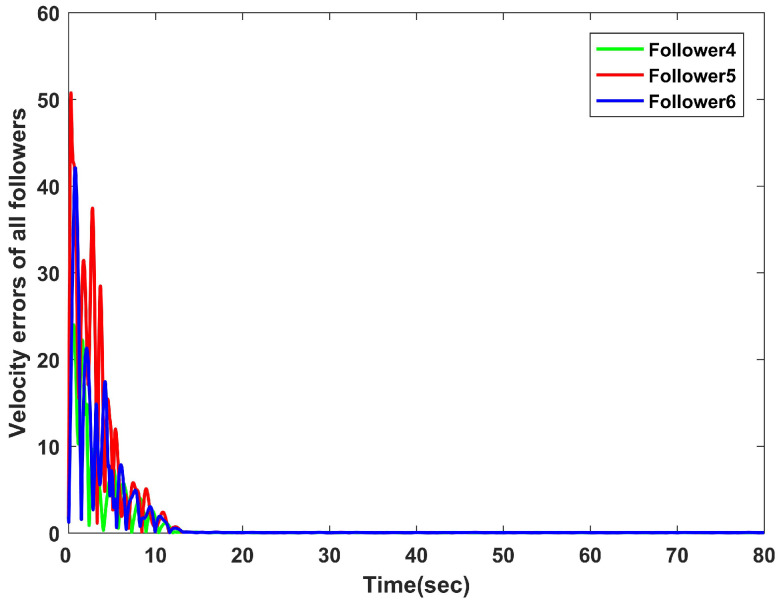
Velocity errors of all followers.

**Figure 8:**
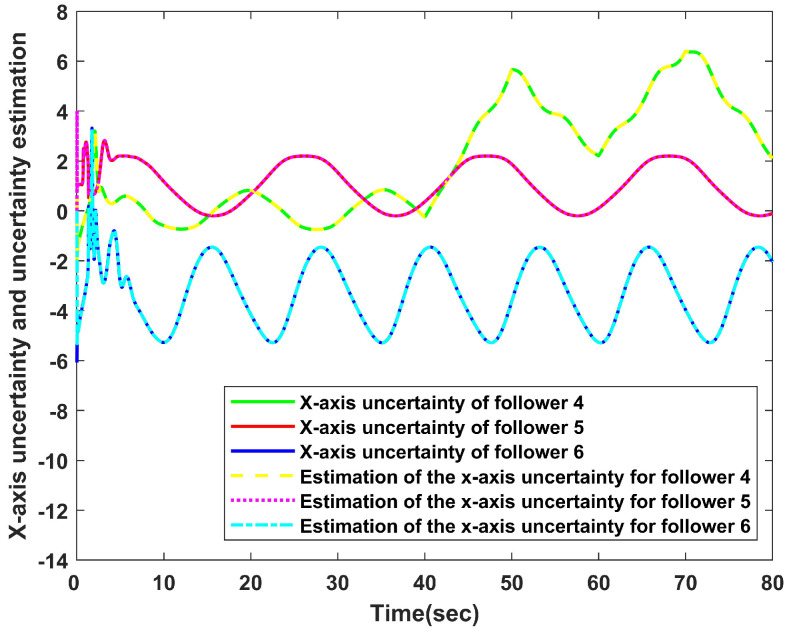
X-axis uncertainty and x-axis uncertainty estimation for all followers.

**Figure 9:**
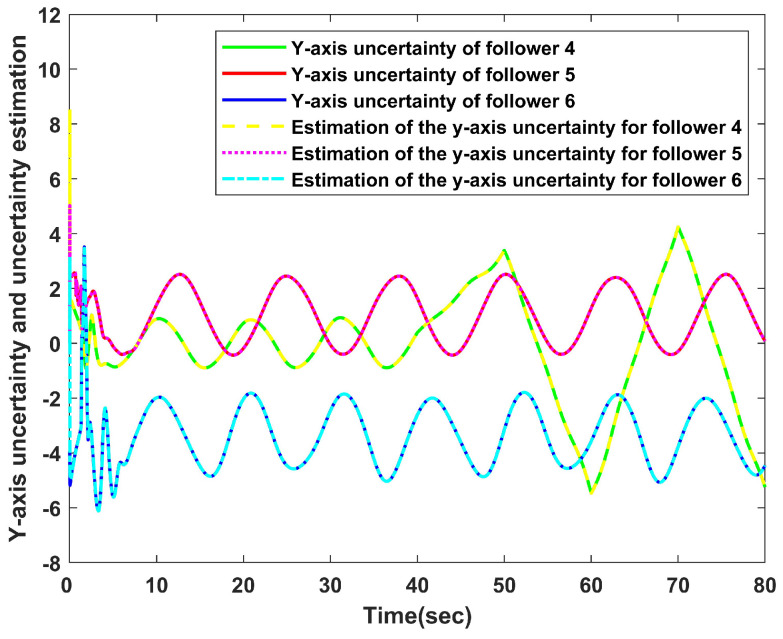
Y-axis uncertainty and y-axis uncertainty estimation for all followers.

**Figure 10:**
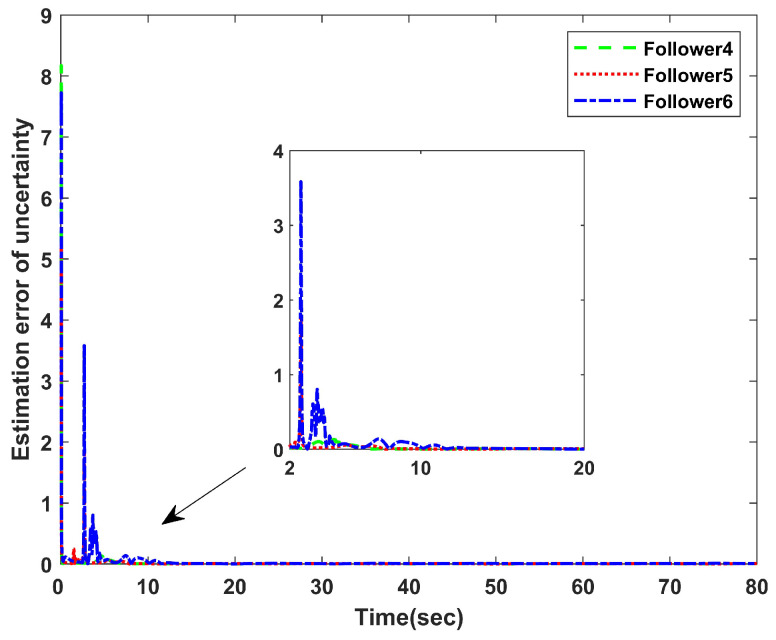
Estimation error of uncertainty.

The bearing error and velocity error of follower *i* are defined as $\xi_{gi}=∥\sum_{j\in N_{i}}\left(g_{ij}-g_{ij}^{*}\right)∥$ and $\xi_{vi}=∥v_{i}-v^{*}∥$, respectively. The uncertainty estimation error of follower *i* is defined as $\xi_{Di}=∥D_{i}-{\hat{D}}_{i}∥$. Figure 4 displays the trajectories of all leaders and all followers. Figure 5 illustrates the bearing error of the follower agents, demonstrating that the bearing error of each follower agent can converge to zero. Figure 6 and Figure 7 illustrate the velocities of the agents and the velocity errors of the follower agents. The uncertainty of follower *i* is represented as $D_{i}={\left[D_{ix},D_{iy}\right]}^{T}$, and the uncertainty estimation is denoted as ${\hat{D}}_{i}={\left[{\hat{D}}_{ix},{\hat{D}}_{iy}\right]}^{T}$. Figure 8 displays the uncertainties $D_{4x}$, $D_{5x}$, $D_{6x}$ of followers, along with their estimated values ${\hat{D}}_{4x}$, ${\hat{D}}_{5x}$, ${\hat{D}}_{6x}$. Figure 9 displays the uncertainties $D_{4y}$, $D_{5y}$, $D_{6y}$ and their estimated uncertainties ${\hat{D}}_{4y}$, ${\hat{D}}_{5y}$, ${\hat{D}}_{6y}$. Figure 10 presents the uncertainty estimation errors of all followers, which indicates that such errors can converge to zero within time $T_{D}$. The results from Figure 4, Figure 5, Figure 6, Figure 7, Figure 8, Figure 9 and Figure 10 indicate that the prescribed-time uncertainty observer (17)–(19) can effectively estimate uncertainties composed of multiplicative and additive faults, and that the bearing-only fault-tolerant formation control law (29) enables all followers to converge to the desired geometric formation with the leaders.

### 5.2. Quantitative Performance Metrics and Parametric Sensitivity

**Figure 11:**
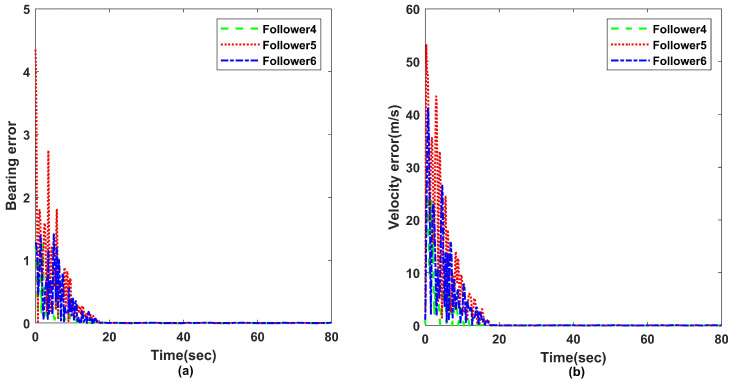
Numerical simulation results under the condition $k_{a}=0.8$, $k_{c}=1.6$, with the remaining parameters of (17)−(19) and (29) set the same as those in Section 5.1: (**a**) bearing errors of the followers; (**b**) velocity errors of the followers.

In this work, the bearing-only fault-tolerant formation control scheme (29) guarantees the asymptotic convergence of both bearing error and velocity error to zero. Parameters $k_{a}$ and $k_{c}$ in control scheme (29) influence the convergence rates of bearing error and velocity error. Let $k_{a}=0.8$ and $k_{c}=1.6$, and the remaining parameter selections for control law (29) are consistent with the parameter settings in Section 5.1. Figure 11 depicts the corresponding numerical simulation results. The results in Figure 11 indicate that the bearing and velocity errors converge to zero at t=20 s. Compared with the simulation results in Section 5.1, the results in Figure 11 illustrate that the convergence rates of the followers’ bearing and velocity errors decrease as the values of $k_{a}$ and $k_{c}$ reduce.

**Figure 12:**
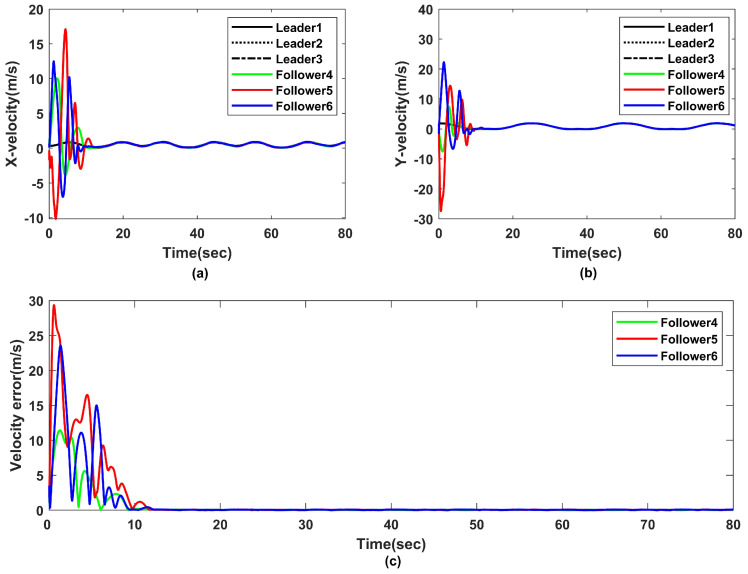
Numerical simulation results under the condition $k_{b}=20$, with the remaining parameters of (17)−(19) and (29) set the same as those in Section 5.1: (**a**) x-axis velocities of all agents; (**b**) y-axis velocities of all agents; (**c**) velocity errors of all followers.

Let $k_{b}=20$, and the remaining parameters of control scheme (29) are identical to those in Section 5.1. Figure 12 shows the velocities of all agents and the velocity errors of all followers. Compared with the simulation results in Figure 6 and Figure 7, the steady-state errors of all followers’ velocities in Figure 12 are significantly reduced. In fact, as the value of $k_{b}$ in control scheme (29) decreases, $∥u_{i}∥$ also declines. Furthermore, the velocity variation rate of each follower is reduced, and the steady-state velocity errors of all followers decrease accordingly.

### 5.3. Comparative Numerical Simulations

**Figure 13:**
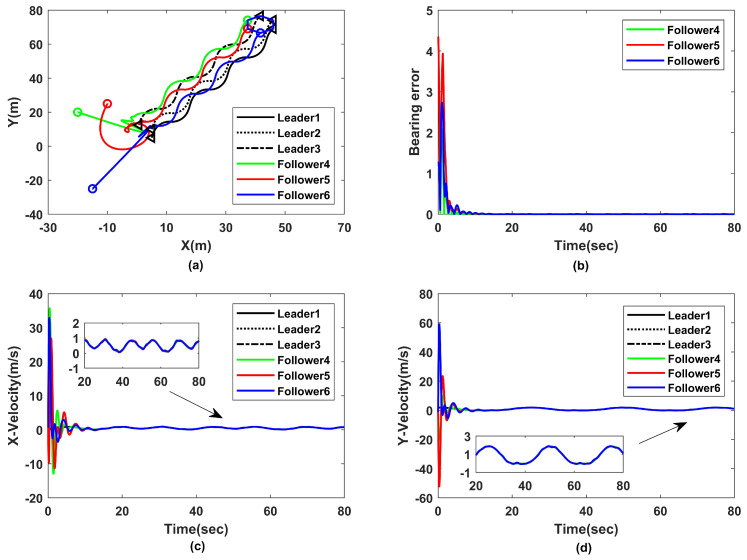
Numerical simulation results of the existing work [32]: (**a**) trajectories of all agents; (**b**) bearing errors of all followers; (**c**) X-velocity of all agents; (**d**) Y-velocity of all agents.

We use the fault-tolerant formation control algorithm relying on bearing measurements proposed in [32] for comparison. The topology of all agents is the same as that shown in Figure 3. The leader’s time-varying velocity $v^{*}\left(t\right)$, followers’ multiplicative faults $\rho_{4}\left(t\right)$, $\rho_{5}\left(t\right)$, $\rho_{6}\left(t\right)$ and additive faults $b_{4}\left(t\right)$, $b_{5}\left(t\right)$, $b_{6}\left(t\right)$ remain the same as the settings in Section 5.1. The parameter settings of the method proposed in [32] are $k_{p}=10$, $k_{v}=2$, $\varepsilon_{v}=1$, $\alpha_{1}=3$, $\alpha_{2}=4$, $\gamma_{1}=1$, $\gamma_{2}=1.3$, $\gamma_{3}=1.6$. Figure 13 shows the numerical simulation results of the method proposed in [32]. The simulation results demonstrate that the bearing errors of all followers converge to zero. Meanwhile, the velocities of the followers become consistent with those of the leaders, and all agents form the preset geometric structure of the target formation. It should be noted that the method proposed in [32] requires each follower to measure both the relative bearing and relative velocity of its neighbors, while the method developed in this work does not need relative velocity measurements among agents. Compared with the method in [32], the approach proposed in this work is more general and applicable.

## 6. Conclusions

This work develops a fault-tolerant control strategy for followers with actuator failures that relies on angle measurements. A predetermined-time uncertainty observer and a fault-tolerant formation control method are designed to achieve the target formation of multi-agent systems. Numerical simulation results show that the uncertainty estimation error converges to zero within the prescribed time, and all agents asymptotically form the predefined geometric configuration of the formation. Future work will focus on reducing the number of leaders and addressing bearing measurement inaccuracy caused by visual sensor chattering. Robust control methods will be developed to improve the stability of multi-agent systems.

## Data Availability

No datasets were generated or analysed during the current study.
